# Long-Tune Natural Logarithmic Wavelength Modulation Spectroscopy for Gas Sensing

**DOI:** 10.3390/s24227365

**Published:** 2024-11-19

**Authors:** Lijuan Lan, Changsheng Zhang, Yibo Wang, Yu Xie, Luheng Wang, Chunhua Yang

**Affiliations:** School of Automation, Central South University, Changsha 410083, China; lijuan.lan@csu.edu.cn (L.L.); cs_zhang323@163.com (C.Z.); yb_wang@csu.edu.cn (Y.W.); 224612190@csu.edu.cn (Y.X.); ychh@csu.edu.cn (C.Y.)

**Keywords:** long-tune spectrum, ln-WMS, background signal, concentration measurement

## Abstract

This article presents a gas sensing method based on long-tune natural logarithmic wavelength modulation spectroscopy (long-tune ln-WMS) and explores means to improve its accuracy. The long-tune spectrum can detect multiple gases with high precision. In ln-WMS, due to the natural logarithm algorithm, the harmonic magnitude which is related to gas concentration would not be affected by the light intensity fluctuations. However, the background signal of the harmonic will become strong and nonlinear in the long-tune spectrum. Three CO_2_ absorption lines and one H_2_O line near 2004 nm are applied to verify the proposed theory. The effects of light intensity, modulation depth, gas concentration, and phase shift on the harmonics are tested separately through both simulations and experiments. The results reveal that our proposed method can always keep the harmonics at their maximum which ensures high measurement precision. Moreover, the background signal only varies with the modulation depth, not the concentration and light intensity. Even the mechanical vibrations cannot disturb the harmonics, which enables the proposed method to be suitable for gas detection in harsh environments, especially for heavy dust and severe mechanical vibrations. The CO_2_ concentration detection results indicate that when the background is eliminated, the accuracy can be achieved with a relative error of below 0.5%, while the error would be greater than 5% with background presence. The proposed long-tune ln-WMS method is effective for trace gas detection (weak absorption) or over-modulation conditions and has potential applications in field inspection.

## 1. Introduction

In situ measurements of CO_2_ emissions at industrial sites is of great significance in controlling carbon emissions and realizing the low-carbon operation of the process industry [1,2,3,4]. Spectrometry-based techniques have been widely used for gas monitoring due to the properties of non-contact, fast response, and so on [5,6,7,8], of which the tunable diode laser absorption spectroscopy combined with wavelength modulation spectroscopy (TDLAS-WMS) is one of the most popular technologies applied in the field measurement [9,10,11,12,13]. The tunable diode laser scans across the absorption line at an extremely small bandwidth and the emitted wavelength is modulated by a frequency of kilohertz or even a megahertz sinusoidal signal. The gas parameters of the absorption line can then be acquired by analyzing the demodulated harmonics. Therefore, TDLAS-WMS achieves a higher signal-to-noise ratio (SNR) than other methodologies [14,15].

Traditional TDLAS-WMS, which is referred to as WMS-2f, uses the second harmonic to obtain the concentration [16,17]. However, this method needs to calibrate the second harmonic to the known gas concentrations. To obtain the absolute concentration using the second harmonic directly, some methods are developed to remove factors from the second harmonic, which could interfere with the concentration calculations (the so-called “calibration-free” methods). However, WMS-2f is only available for weak absorption with an absorbance of less than 5% [18]. The calibration-free methods mainly include “2f/1f”, “nf/1f”, “nf/0f”, and so on. Nonetheless, no matter which calibration-free method is used, its application is always limited by the absorbance range. For example, the calibration-free “nf/0f” method can ensure the measurement accuracy with absorbance below 2% [19,20]. The calibration-free “2f/1f” is used for gas detection with absorbance below 10% when the first-order Fourier Expansion is used to approximate the Beer–Lambert law [21,22]. The absorbance can be extended to 30% when using the second-order Fourier Expansion [23]. Even so, the calibration-free methods still cannot meet the needs of CO_2_ emission detection at industrial sites, due to the large varying CO_2_ concentrations in the open air of the process industry [24,25]. Additionally, in field applications, the laser light intensity, a parameter of harmonics, is easily disturbed by harsh environments, such as dust and mechanical vibrations, which renders the calibration-free methods ineffective.

The long-tune spectrum allows for the simultaneous detection of multiple gases or multiple absorption lines of the same gas; thus, it can enhance the measurement precision and anti-interference ability [19,26]. Moreover, the multiple absorption lines enable us to determine the laser-tuning parameters and the scanning wavelength using the positions of absorption lines. Natural logarithmic WMS (ln-WMS) is proposed for operating WMS-2f in any absorbance since the laser light intensity is filtered out in the demodulation process [27]. Therefore, when ln-WMS is combined with the long-tune spectrum, it is expected to conduct calibration-free measurements of the gas concentration in any absorbance. However, according to our explorations, in long-tune ln-WMS, the background signal induced by the residual amplitude modulation (RAM) is different from traditional WMS in that the background signal would be enhanced to a nonlinear curve. It cannot be neglected and is required to be eliminated, especially for weak absorbance or over-modulations. Currently, there is limited research on removing the background from the harmonic. Li et al. [27] used a separated non-absorbance laser beam to search the laser light phase and set the background of the first harmonic to zero. However, the method can only zeroize the background of odd harmonics, while the even harmonics still retain the background signal at its maximum. Moreover, the harmonic amplitudes are restricted by the sin/cos phase shift, resulting in poor measurement precision. Upadhyay et al. proposed a calibration-free second harmonic extraction technique to eliminate RAM signals unrelated to absorption [28]. However, it is used for conventional WMS with a single absorption line. We used an FFT algorithm to detect the phase shift between the wavelength and intensity modulation and used the detected phase shift for RAM elimination in the long-tune spectrum [20], which is also only promoted for traditional WMS.

Based on previous studies, this article focuses on the long-tune ln-WMS method for gas sensing and studies the way to enhance measurement accuracy by eliminating the background signal in harmonics. Three CO_2_ absorption lines and one H_2_O absorption line near 2004 nm are utilized for simulating and testing the harmonics. The influences of light intensity, modulation depth, gas concentration, and modulation frequency (phase shift) on the background and amplitude of harmonics are investigated in both simulations and experiments. The rest of this article is organized in the following sections. Section 2 presents the formula of harmonics based on ln-WMS. Section 3 simulates the impacts of light intensity, modulation depth, and gas concentration on the harmonic. Section 4 introduces the experimental setup. Afterward, the verification experiments and gas sensing are taken in Section 5. Conclusions are finally given in Section 6.

## 2. Theory of ln-WMS

According to the Beer–Lambert law, a laser light with light intensity $I_{0}\left(t\right)$ will be absorbed by gas molecules at a specific wavelength after passing through the measured medium, and the light intensity $I_{\tau }\left(t\right)$ of the transmitted laser can be described as

(1) $$I_{\tau }\left(t\right)=I_{0}\left(t\right)\exp\left[-PS\left(T\right)CL\varphi \left(v\right)\right],$$

where *P* [atm], S(T) [cm^−2^/atm], *C*, *L* [cm], *v* [cm^−1^], and φ(v) [cm] are the gas pressure, absorption line strength, concentration, absorbing light path length, wavenumber, and absorption line profile, respectively. Wavelength modulation spectroscopy (WMS) uses a low-frequency ramp signal and a high-frequency sinusoidal signal to modulate the laser simultaneously, and the instantaneous wavenumber v(t) and original light intensity $I_{0}\left(t\right)$ can be expressed as [27,29]

(2) $$\left\{\begin{array}{c} \begin{array}{cc} v(t) & =\bar{v}+a\cos\theta \\ I_{0}\left(t\right) & =\bar{I_{0}}+\sum_{k=1}^{\infty }I_{k}\cos\left(k\theta +\psi_{k}\right)=\bar{I_{0}}\left(1+\sum_{k=1}^{\infty }i_{k}\cos\left(k\theta +\psi_{k}\right)\right), \end{array} \end{array}\right.$$

where $\bar{v}$ [cm^−1^] is the central wavenumber, which is the wavenumber of the central laser emission implemented by the ramp signal; *a* [cm^−1^] is the modulation depth; θ=ωt [rad] is the radian; ω [rad/s] is the modulation angular frequency; $\bar{I_{0}}$ is the central light intensity, which is also determined by the ramp signal; $I_{k}$ is the *k*-th order sinusoidal modulation light intensity; and $\psi_{k}$ is the phase shift between wavelength modulation and the *k*-th order light intensity modulation. Defined: $i_{k}=I_{k}/\bar{I_{0}}$, which can be considered the relative light intensities of the orders of modulation light intensity to the central light intensity. Specially, $i_{k}$ is only determined by the modulation depth *a*. Thus, they would be constant items at a fixed wavelength when *a* is fixed. We will discuss $i_{k}$ in the following subsections (Section 3.2 and Section 5.2).

Substituting Equation (2) into Equation (1) and taking the natural logarithm of both sides of Equation (1),

(3) $$\begin{array}{cc} \ln[I_{\tau }\left(t\right)] & =\ln[I_{0}\left(t\right)]-PS\left(T\right)CL\varphi \left(v\right)\\ \; & =\ln\bar{I_{0}}+\ln\left(1+\sum_{k=1}^{\infty }i_{k}\cos\left(k\theta +\psi_{k}\right)\right)-PS\left(T\right)CL\sum_{k=0}^{\infty }H_{k}\cos k\theta , \end{array}$$

where $H_{k}$ is the *k*-th order Fourier coefficient of φ(v). Since the higher order (k≥3) sinusoidal modulation strength is too small to consider compared to the first two orders, $\ln[I_{0}\left(t\right)]$ can be simplified as

(4) $$\ln\left[I_{0}\left(t\right)\right]\approx \ln\bar{I_{0}}+\ln\left[1+i_{1}\cos\left(\theta +\psi_{1}\right)+i_{2}\cos\left(2\theta +\psi_{2}\right)\right].$$

Since $i_{1}$ and $i_{2}$ are sufficiently smaller than one, we can further simplify Equation (4) according to Taylor Expansion:

(5) $$\ln\left[I_{0}\left(t\right)\right]\approx \ln\bar{I_{0}}-0.25i_{1}^{2}+i_{1}\cos\left(\theta +\psi_{1}\right)+i_{2}\cos\left(2\theta +\psi_{2}\right)-0.25i_{1}^{2}\cos\left(2\theta +2\psi_{1}\right).$$

Then, substituting Equation (5) into Equation (3) and multiplying $\ln[I_{\tau }\left(t\right)]$ with the corresponding reference signals, the harmonic signals can be obtained through a low-pass filter. The following will discuss the influence of the reference signal phase on the harmonics. As described in Ref. [27], a non-absorbance light path is installed to seek the reference signal phase to make the first harmonic of non-absorbance minimum, which is equivalent to setting the reference signal as $\cos(\theta +\psi_{1}+\pi /2)$ and $\cos(2\theta +2\psi_{1}+\pi )$ for the first and second harmonics, and then the harmonics can be obtained:

(6) $$\left\{\begin{array}{c} \begin{array}{cc} S_{1f}^{\mathrm{LN}} & =PS\left(T\right)CLH_{1}\sin\psi_{1}\\ S_{2f}^{\mathrm{LN}} & =-i_{2}\cos\left(2\psi_{1}-\psi_{2}\right)+0.25i_{1}^{2}+PS\left(T\right)CLH_{2}\cos2\psi_{1}. \end{array} \end{array}\right.$$

The above theory is known as the natural logarithmic WMS (ln-WMS) method. It can be seen that the derived results contain two parts: the $i_{k}$ items are considered the background signal and the $PS\left(T\right)CLH_{k}$ items are the harmonics associated with gas concentration. Since the harmonic signal is linear to gas concentration, the ln-WMS method can be used for gas concentration monitoring at any large absorbance. However, we can learn from Equation (6) that although the reference signal phase can help the odd harmonics eliminate the background signal, the even harmonics always maintain the maximum background signal. Meanwhile, the target harmonic signals are constrained by the sine/cosine phase shift ($\psi_{k}$) and thus cannot keep the optimum. The influence of the phase shift ($\psi_{k}$) will be discussed in Section 5.3.

On the other hand, when the reference signals are set as cosθ and cos2θ, the first and second harmonics can be

(7) $$\left\{\begin{array}{c} S_{1f}^{\ln}=i_{1}\cos\psi_{1}-PS\left(T\right)CLH_{1}\\ S_{2f}^{\ln}=i_{2}\cos\psi_{2}-0.25i_{1}^{2}\cos2\psi_{1}-PS\left(T\right)CLH_{2}. \end{array}\right.$$

Unlike Equation (6), Equation (7) maximizes the harmonic signals; thus, the optimal harmonics can be applied for high-precision gas sensing. We will use the reference signal and demodulation method as expressed in Equation (7). In measurement, the background signal is usually treated as a constant value or ignored. Nevertheless, in the long-tune spectrum, the $i_{k}$ items would vary with the wavelength scanning. Specially, the strong first-order light intensity, $i_{1}$, is contained in the second harmonic as a square component ($i_{1}^{2}$), resulting in a powerful and nonlinear background signal of the second harmonic, which makes the removal of the background signal and the concentration calculation difficult.

## 3. Simulation of Long-Tune ln-WMS

In this section, the harmonic and its background obtained by long-tune ln-WMS is simulated. The long-tune spectrum is applied for gas sensing since it scans across several absorption lines and can determine the tuning parameters and detect multiple gas concentrations simultaneously. Meanwhile, with more spectral lines used for detection, it can effectively reduce noise interference and improve accuracy. However, the background signal in long-tune harmonics will be enhanced and cannot be neglected due to the RAM becoming distinct, especially for the ln-WMS method. The CO_2_ and H_2_O absorption lines near 2004 nm are utilized for simulating long-tune ln-WMS. The absorption spectroscopic parameters of the selected transitions are downloaded from the HITRAN Databases [30] and listed in Table 1. For the simulation, the atmosphere conditions are set as a pressure of 1 atm, a temperature of 296 K, and an absorbing light path length of 30 cm, respectively. The environmental parameters, Lorentz broadening and Gaussian broadening, and the discrete spectral data will be combined to calculate a continuous absorption spectrum. Then, the high-frequency sinusoidal signal is added in wavelength scanning and the transmitted light intensity signal is converted according to Equation (1). Finally, the transmitted light intensity signal is applied for harmonic demodulation, and the harmonic signals of consecutive multiple absorption spectra are obtained. In the following subsections, we will investigate the influences of laser light intensity, modulation depth, and gas concentration on the second harmonic, respectively.

### 3.1. Light Intensity Attenuation

**Figure 1:**
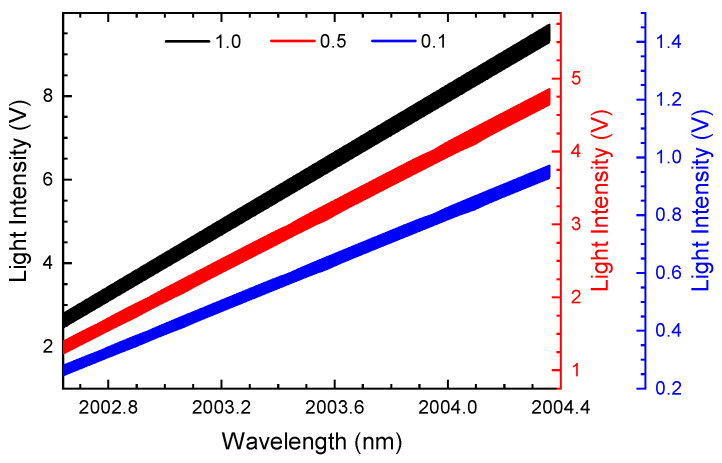
The transmitted light intensity attenuates (the parameters in the figure are the attenuation indices); the concentrations of CO_2_ and H_2_O are 0.001 and 0.03, respectively; the modulation depth remains at 0.168 cm^−1^.

**Figure 2:**
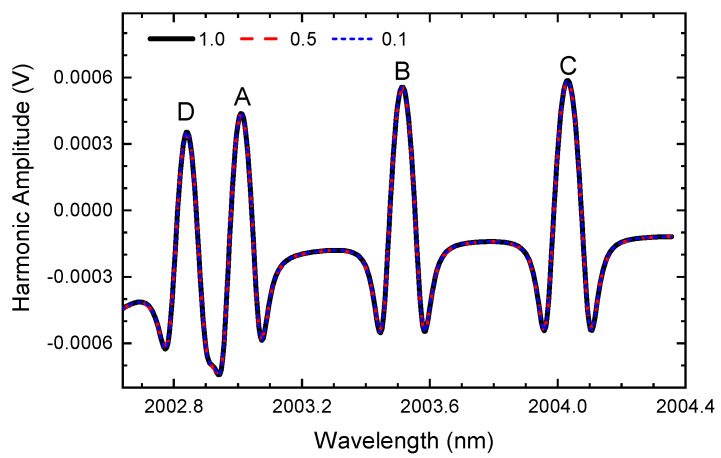
The second harmonic signal obtained from the transmitted light intensity of Figure 1 using the ln-WMS method, symbols A–D are the absorption lines as listed in Table 1.

In field measurements, affected by dust interference and optical path drift, the transmitted laser light intensity received by the PD is frequently fluctuating or time-varying. Since the harmonic signals demodulated by conventional WMS contain the laser light intensities, the harmonic amplitudes are strongly affected by the light fluctuation, which results in calibration failure in applications. For the ln-WMS method, we simulate the light intensity fluctuating by adding an attenuation index, and the attenuated spectrum signals are shown in Figure 1, where the attenuation indices are set as 1.0, 0.5, and 0.1, respectively. The gas concentrations of CO_2_ and H_2_O are 0.001 (1000 ppm) and 0.03, respectively. The modulation depth is set at 0.168 cm^−1^ (modulation index, m≈2.2), while the other gas parameters remain the same. The second harmonics demodulated by the ln-WMS method are demonstrated in Figure 2.

Different from what was expected in conventional WMS, as shown in Figure 2, the harmonic signals obtained from the ln-WMS method are highly consistent. The amplitudes of the demodulated signals remain the same when the laser light intensity is attenuating. The simulation results easily prove the conclusion in Equation (7); that is, the harmonic signal demodulated by the ln-WMS method can exclude the central light intensity ($\bar{I_{0}}$). Therefore, ln-WMS can effectively overcome the problem of light intensity fluctuations without the process of calibrating the second harmonic signal by the zeroth harmonic (nf/0f) or the first harmonic (nf/1f). The advantage makes it especially suitable for gas monitoring in harsh environments where the light intensity usually fluctuates violently. However, it should be noticed that the background signal of the second harmonic varies in the long tune, and we will discuss this phenomenon in the following subsections.

### 3.2. Modulation Depth

**Figure 3:**
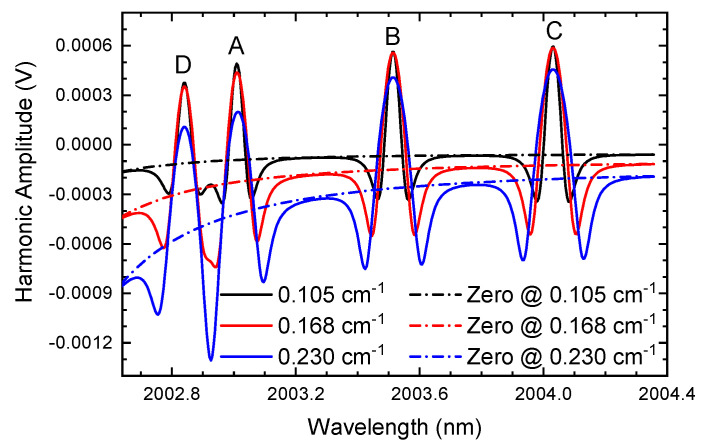
The demodulated second harmonics when there is gas absorption (the concentrations of CO_2_ and H_2_O are 0.001 and 0.03, respectively) and no gas absorption (the modulation depths vary from 0.105 cm^−1^ to 0.230 cm^−1^).

Subsequently, the modulation depth gradually increases while the gas concentrations of CO_2_ and H_2_O are fixed at 0.001 and 0.03, respectively, and the laser light intensity also remains the same. Three modulation depths, low modulation with 0.105 cm^−1^ (m≈1.4), medium modulation with 0.168 cm^−1^ (m≈2.2), and high modulation with 0.230 cm^−1^ (m≈3.0), are computed separately, and the demodulated second harmonics according to ln-WMS are demonstrated in Figure 3. For comparison, the situations with no gas absorption with the corresponding modulation depths are added as dotted curves in the graph.

**Figure 4:**
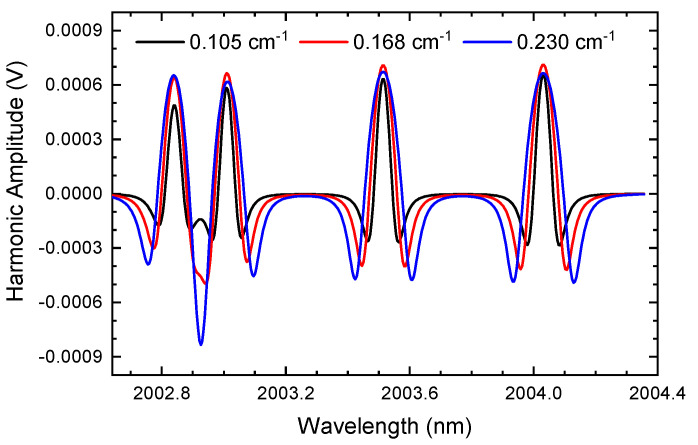
The revised second harmonics of Figure 3 when subtracted by the background (the concentrations of CO_2_ and H_2_O are 0.001 and 0.03, respectively, and the modulation depths vary from 0.105 cm^−1^ to 0.230 cm^−1^).

From Figure 3, we can find that at a specific modulation depth, the baseline of the second harmonic is a distinct curve rather than a definite value or a straight line as in traditional WMS. The background at the beginning of the scanning (left part) is larger than the ending edge, making the harmonic at the left side deformed. In the background of the second harmonic, the square of the first-order relative intensity ($i_{1}^{2}$) (Equation (7)) is dominant in the signal, leading to the harmonic deformity. The background signal is enhanced when the modulation depth (*a*) increases, even causing serious distortion of the harmonics. This is because the relative intensities are positively correlated with the modulation depth. When the modulation depth increases, $i_{k}$ increases and the background strengthens. The results can be observed more obviously when there is no gas absorption (dotted curves). When the harmonics (solid lines) subtract the corresponding curves without gas absorption (dotted lines), the corrected signals are obtained as in Figure 4. It can be seen that the curves restore the harmonic waveform in a high quality. The revised harmonics exclude the background signal as the harmonic amplitude at the gas-free absorption band remains at zero. Thus, the curves without absorption can be regarded as the background signal.

### 3.3. Gas Concentration

**Figure 5:**
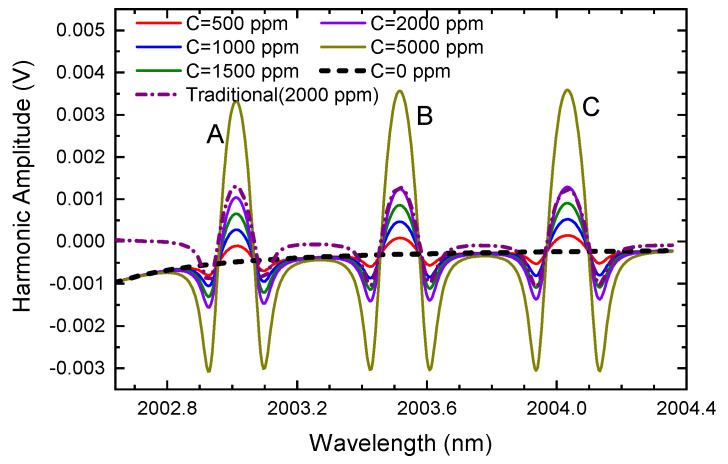
The second harmonic when the concentration varies and the modulation amplitude remains at 0.230 cm^−1^ (only CO_2_ absorption lines are considered as CO_2_ concentration increases from 0 to 5000 ppm; the purple dash–dotted line is the harmonic extracted by traditional WMS with CO_2_ concentration of 2000 ppm).

Finally, the effect of gas concentration on the ln-WMS harmonic is explored. In the simulation, the modulation depth is fixed at 0.230 cm^−1^ (m≈3.0) while the CO_2_ concentration is increased from 0 ppm to 5000 ppm (0.5%). The other parameters and conditions remain the same. The demodulation results are demonstrated in Figure 5.

Different from Figure 3, where the background changes as the modulation depth varies, the background signals in Figure 5 stay the same when the CO_2_ concentration increases. That is to say, the gas concentration has no effect on the background signal, which is consistent with the theoretical analysis in Equation (7). However, it should be noted that compared to the theoretical harmonic or second harmonic extracted using traditional methods (as the purple dash–dotted line shown in the graph), which have relatively flat baselines (stable around 0), the second harmonics obtained using the ln-WMS method exhibit sloped baselines at different concentrations. Therefore, it can be considered that the background signal has a more significant impact on the second harmonics extracted by the ln-WMS method, especially in long-tune situations. When in the trace gas monitoring or in the over-modulation, the background signal becomes too strong to be ignored. The good news is that it does not change with concentration, so we can determine the background signal at a known modulation depth. Then, the modulation depth should be fixed to follow gas detection. The previously determined background signal is applied for background elimination. Moreover, we should find that as the concentration increases, the amplitude of the second harmonic increases while the influence of the background decreases. For example, at 5000 ppm, the background of Line A is about one-seventh of the harmonic peak, and the proportions of the other lines are much smaller. Therefore, in the following discussions and experiments, we will only consider a CO_2_ concentration of below 5000 ppm.

In summary, unlike traditional WMS, the harmonic signals of long-tune ln-WMS have stronger backgrounds with a nonlinear curve. Fortunately, we can learn that the background signal in the ln-WMS method can only be affected by the modulation depth, not the gas parameters or the light intensities, although its formula is expressed by the variables of light intensities. In applications, the background signal can be eliminated according to this feature: determine the background at a specific modulation depth prior to the measurement and then apply it for the background subtraction in the actual measurement. The background elimination method can be useful when the background is too strong to be neglected, especially when the gas concentration is low (weak absorbance) or in over-modulation situations.

## 4. Experimental Foundation

**Figure 6:**
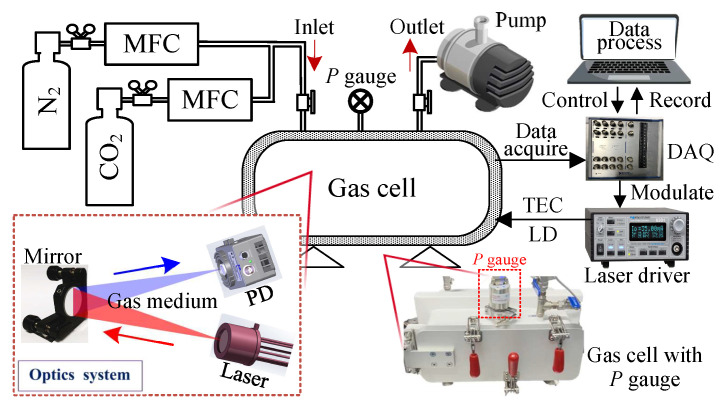
Schematic of the ln-WMS measurement system (lower right: the steel-sealed gas cell with pressure gauge; lower left: the optical system).

To confirm the gas sensing method based on long-tune ln-WMS, experiments are carried out using three CO_2_ absorption lines and one H_2_O line near 2004 nm. The schematic diagram of the ln-WMS measurement system is shown in Figure 6. A steel-sealed gas cell is evacuated by a vacuum gas pump with an ultimate pressure of 20 Pa and then filled with a CO_2_/N_2_ mixture. The standard gases of CO_2_ and pure N_2_ with concentrations of 0.5% (5000 ppm) and 99.99%, respectively, running into the gas cell are controlled by flow controllers (MFCs) to ensure the concentration of the mixture. A pressure gauge (PVG 550, Infitech, Shanghai, China) is set to measure the gas pressure inside the cell, which is used to determine whether the gas cell has reached a vacuum state (the gas has been almost completely evacuated) or is filled with gas at 1.0 atm. After the gas cell is pumped to the ultimate pressure, the pressure inside can be maintained below 100 ± 2 Pa within 30 h. The whole detection light path, including the laser, photodetector (PD, PDA10DTEC, Thorlabs, Newton, MA, USA), and concave reflector (Mirror, CM254-075-G01, Thorlabs), is arranged in the gas cell. As the optical system shown in the enlarged image in the lower left corner, a VCSEL (vertical cavity surface emitting laser, Vertilas GmbH, Munich, Germany ) laser, whose temperature and current are controlled by a laser driver (Arroyo Instruments 6301, San Luis Obispo, CA, USA), works as the laser source. The emitted laser travels through the measured gas medium and is reflected by the mirror with a focus length of 75 mm. The absorbing light path length is 30 cm. The absorbed light signal is received by the PD and then converted into an electrical signal. Afterward, the detected signal is recorded by a high-speed data acquisition card (DAQ, NI USB-6361, Austin, TX, USA) and sent to the computer for data processing. Moreover, the DAQ also serves as a signal generator to produce a low-frequency ramp signal and a high-frequency sine signal to modulate the laser wavelength. The aviation connectors are utilized to connect signals of controlling and receiving inside and outside the gas cell.

A data processing system is built in LabVIEW software, (2020 SP1). It contains three parts: signal control, harmonic demodulation, and concentration calculation. In signal control, a low-frequency ramp signal (520 mV, 10 Hz) is applied to scan the light wavelength in long tune, a high-frequency sinusoidal signal (e.g., 6.0 kHz) is used to modulate the laser wavelength, and the amplitude of the sine signal (the modulation voltage) can be adjusted. The received signal is demodulated by a digital lock-in amplifier (LIA) in the demodulation unit. In LIA, an FFT unit is set to detect the phase shift between the wavelength and intensity modulation, and then the detected phase shift is utilized to set the reference signal phase as expressed in Equation (7). For the ln-WMS method, the received signal (transmitted laser intensity) is taken as the natural logarithm before demodulation. The gas concentration is finally calculated based on the demodulated harmonics [19].

## 5. Experimental Results and Analysis

### 5.1. Light Intensity Vibration

**Figure 7:**
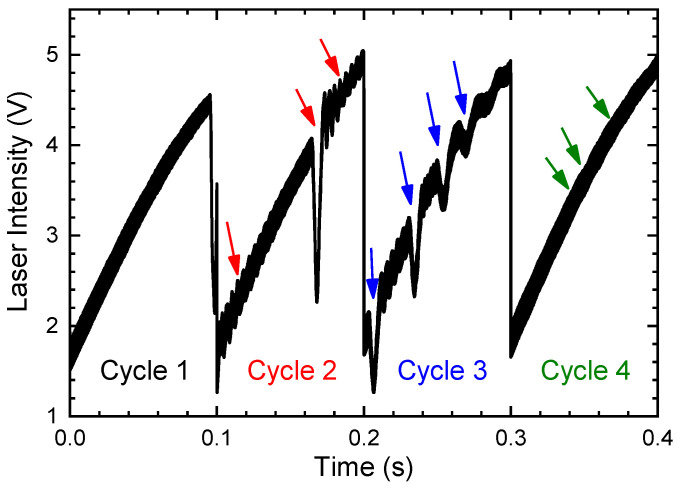
Spectral signal received from PD when there are vibrations in the measurement system (the measured medium is the air in the laboratory with concentrations of CO_2_ and H_2_O of about 1000 ppm and 0.6%, respectively, and the modulation voltage is 50 mV (with modulation depth of 0.250 cm^−1^)). The arrows indicate the fluctuations in the spectral signals.

Similar to the above simulations, the effects of light intensity, modulation voltage (depth), gas concentration, and phase shift are tested in the experiments. All the experiments are conducted at room temperature (296 ± 1 K) with a pressure of 1.0 atm (±50 Pa). In the very beginning, the light intensity is investigated. To obtain different light intensities, an irregular vibration is loaded on the optical system. The optical system and the mechanical vibration device are deployed in the air of the laboratory. During the vibrations, the absorption light path is still fixed at the same light path length, and only the receiving spectral light intensity would fluctuate. The spectral signal received by the PD is demonstrated in Figure 7. We can see that the four scanning cycles of the spectral signal are quite different. In the first cycle (Cycle 1), it can be served as the normal signal without any disturbance. The remaining three cycles can be regarded as being disturbed by three different vibrations: fast vibration (Cycle 2), low-frequency strong vibration (Cycle 3), and micro-vibration (Cycle 4), respectively. The vibrations are marked as three different color arrows in the figure, some of which are easily mistaken to be the absorption lines. Moreover, we can find that the light intensity in the last three cycles is slightly enhanced when compared with the first cycle.

**Figure 8:**
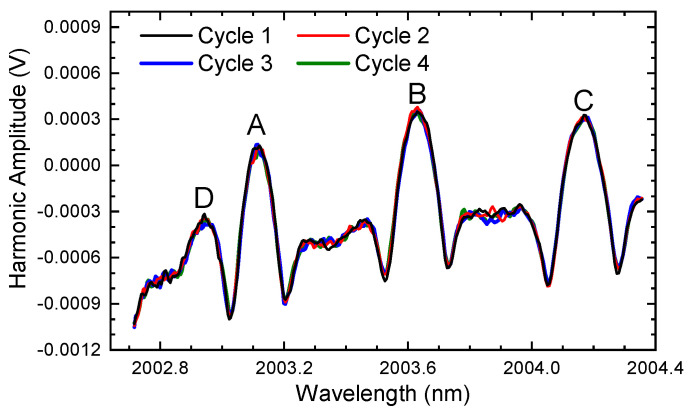
The second harmonic demodulated from Figure 7 based on ln-WMS.

When the four-cycle spectral signals are sent for demodulation using the ln-WMS method, the harmonic signals are obtained as demonstrated in Figure 8: all the harmonics of the four cycles have high consistency without any drift. Similar to the results shown in Section 3.1, the light intensity attenuation or enhancement has no impact on the harmonic under ln-WMS theory (Cycle 2, 3, and 4 compared with Cycle 1). Moreover, the experimental results indicated that mechanical vibrations also cannot have any interference on the demodulated results, regardless of whether it is high or low frequency or has strong or weak vibrations. It produces the powerful anti-interference ability of the ln-WMS method. Therefore, we can conclude that the ln-WMS method can always maintain a high detection performance, especially in harsh environments when there is heavy dust (causing light intensity attenuation) or mechanical vibrations (resulting in light intensity fluctuations).

### 5.2. Modulation Voltage

**Figure 9:**
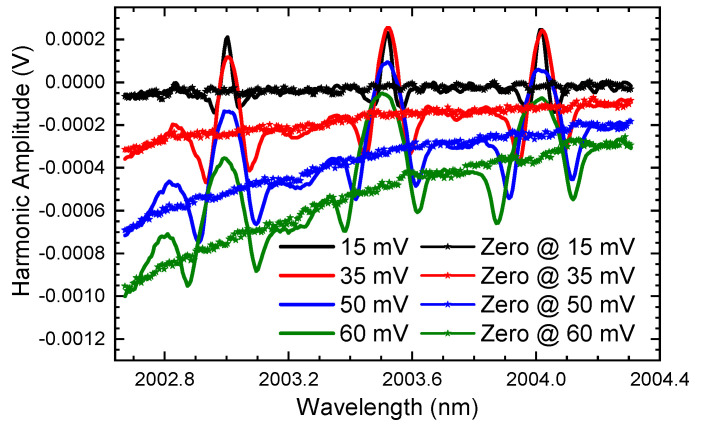
The demodulated second harmonic when CO_2_ concentration is fixed at 500 ppm and the modulation voltage increases from 15 mV to 60 mV (the point curves are harmonics at corresponding modulation voltages without gas absorption).

Secondly, the modulation depth (voltage) is studied. To ensure that the gas concentration remains unchanged during the experiment, we lock the optical system in the gas cell. By evacuating air and injecting N_2_, the gas concentration and pressure inside the cell are controlled at fixed values. The modulation voltage of the high-frequency sinusoidal signal increased from 15 mV to 60 mV. To better display the background signal, two CO_2_ concentrations of 500 ppm and 0 ppm (zero absorption, filled with N_2_) are measured separately. The results of the second harmonic signals with different modulation voltages and different concentrations are shown in Figure 9.

We can find that the obtained second harmonics in Figure 9 are consistent with the simulation results without any suspense. That is, the background of the second harmonic will be enhanced when the modulation depth (voltage) increases and the curve of the background overlaps with that at zero absorption, which verifies the correctness of the theory.

### 5.3. Phase Shift

In WMS, the phase shift between wavelength modulation and light intensity modulation ($\psi_{k}$) will gradually increase when the modulation frequency becomes higher. Among them, the first-order phase shift ($\psi_{1}$) plays the most important role in the harmonics since the first-order relative intensity ($i_{1}$) dominates in the harmonic formulas. Hence, we only focus on $\psi_{1}$ in the following analysis. The phase shift is measured using an FFT module in LabVIEW software when its detecting frequency is set as the modulation frequency. In our experimental system, the phase shift $\psi_{1}$ changes from 6.7° to 20° when the modulation frequency is adjusted from 6.0 kHz to 30.0 kHz. Limited by the experimental conditions, a higher frequency has not yet been tested.

**Figure 10:**
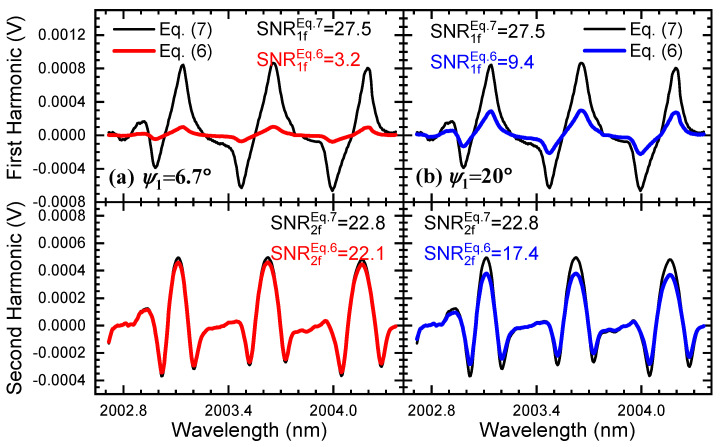
The demodulated first and second harmonics using different methods when phase shift $\psi_{1}$ varies from 6.7° (**a**) to 20° (**b**), where the background signals have all been eliminated.

Figure 10 exhibits the amplitudes and SNR of the first and second harmonics obtained by using the methods expressed in Equation (6) and Equation (7) when the phase shift changes from 6.7° to 20°. In the graphs, the background signals of the first and second harmonics were eliminated beforehand, and only the magnitudes of the harmonic signals are compared. It is easy to see that the magnitudes of the harmonics cannot reach their maximum when using the former method in Ref. [27]. Furthermore, the first and second harmonics are unable to maintain their optimum simultaneously since they are separately constrained by the sine and cosine of the phase shift according to Equation (6). On the contrary, the outcomes of Equation (7) in Figure 10 indicate that the demodulation and background elimination methods proposed in this article always help to obtain the maximum of target harmonic signals. The larger magnitude of harmonics helps to keep a better SNR of the signal; therefore, it can be expected to obtain a higher precision result in gas sensing.

### 5.4. Concentration and Its Measurement

**Figure 11:**
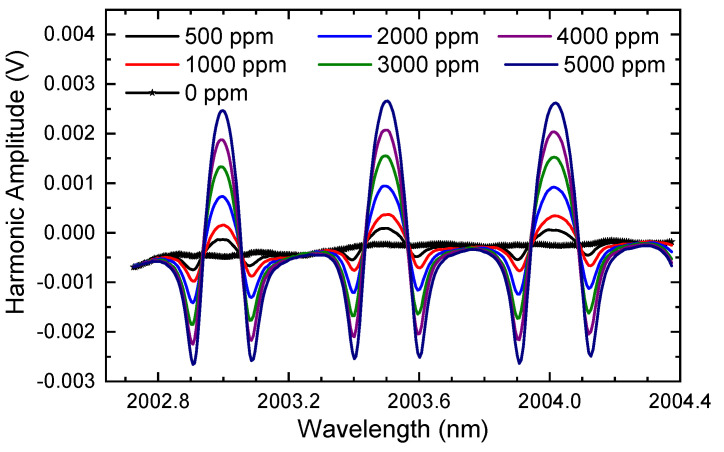
The demodulated second harmonic when the modulation voltage is maintained at 50 mV and the CO_2_ concentration increased from 0 ppm to 5000 ppm.

Subsequently, the modulation voltage is fixed at 50 mV and the impact of gas concentration is tested. Unlike the previous subsections, the gas cell is firstly almost evacuated of the air inside and then injected with pure N_2_ (99.99%) to create an environment without gas molecules of H_2_O and CO_2_ (0 ppm). After that, the CO_2_ concentration is gradually increased from 0 ppm to 5000 ppm by controlling the flow controllers (MFCs) of the standard CO_2_ and N_2_. During the detection, the pressure in the gas cell is maintained at 1.0 atm. The spectral signals are sent for demodulation, and the results are illustrated in Figure 11. It can be seen that the harmonic amplitude increases as the concentration increases. However, the background signal of the harmonic stays the same, indicating similar outcomes as in Figure 5.

**Figure 12:**
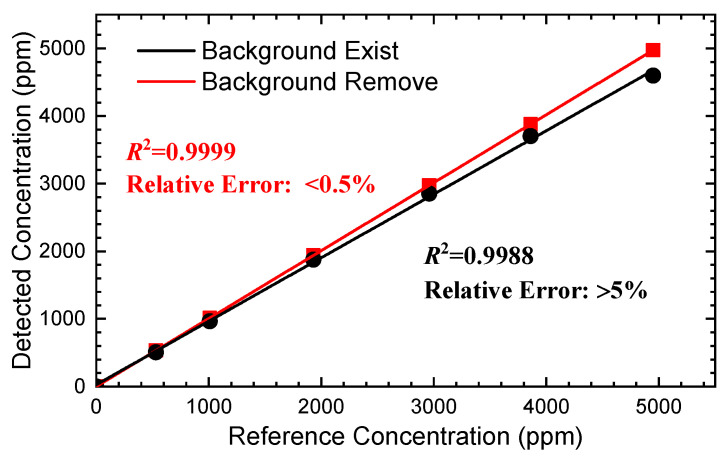
Concentration measurement results when the background signal is eliminated or not (the harmonics are from Figure 11).

Finally, the curve-fitting algorithm is applied to calculate the CO_2_ concentrations by using the long-tune second harmonics, as shown in Figure 11. In the calculation, the background can be eliminated by subtracting the harmonic when the gas concentration is zero (as in the line–star curve in Figure 11) or by using a slope and an offset in the curve-fitting algorithm [19]. The calculation results are presented in Figure 12.

**Figure 13:**
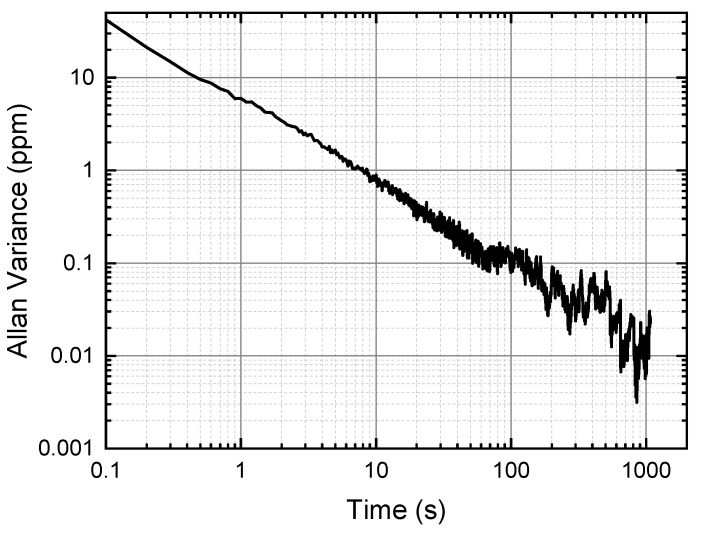
Allan variance analysis of the proposed method.

We can learn from Figure 12 that when the background signal is eliminated, the concentration calculation is able to obtain a better result with a relative error of below 0.5%. In contrast, if the background exists, the measurement concentrations would be lower than the right results with a relative error higher than 5%. Moreover, the measured concentrations illustrate a better linear-fitting result with an R-square of 0.9999 when the background is subtracted. A long-term concentration measurement is conducted according to the proposed method (the concentration is fixed), and the concentration results are analyzed by the Allan variance analysis [31]. The analysis is shown in Figure 13. We can easily find that the measurement captures a fine outcome with a variance of below 1 ppm with 10s averaging time and about 0.1 ppm with 1 min averaging time. The experimental results demonstrate that the background signal has a significant impact on the harmonic in long-tune ln-WMS, which presents a distinct nonlinear curve in the wide scanning wavelength range. Especially, in the trace gas measurement (due to the weak absorbance) or in the over-modulation situations, the strong background signal accounts for a large proportion of the demodulation results, which seriously affects the detection accuracy and precision. Therefore, when the background is eliminated, long-tune ln-WMS can obtain expected gas sensing results.

## 6. Conclusions

This paper investigates the gas sensing approach based on long-tune ln-WMS and searches the means to improve measurement accuracy by eliminating the background signal in harmonics. Three CO_2_ absorption lines and one H_2_O absorption line near 2004 nm are used for the simulation and validation of the harmonic signals of the proposed method. A measurement system with a sealed gas cell and a fixed optical path is built for experimental verification. The impacts of light intensity, modulation depth, gas concentration, and phase shift (modulation frequency) on the harmonic signal are tested separately in both simulations and experiments. The outcomes demonstrate that the proposed method is able to maintain all harmonics at their highest amplitudes, which can further guarantee a high precision in gas detection. In addition, the simulation and experimental results reveal that the background of the harmonics would be enhanced and become a distinct nonlinear curve in the long-tune spectrum when compared to conventional WMS. However, the background signal can only be strengthened by increasing the modulation depth, while the variation in light intensity and gas concentration plays no role in the background. Due to the natural logarithm algorithm, even the distortion of transmitted laser signals caused by mechanical vibrations cannot disturb the background and the harmonic signals. Therefore, long-tune ln-WMS has a superior anti-interference ability and is suitable for gas detection in harsh environments, especially for areas with heavy dust and severe mechanical vibrations. According to these, the background signal can be determined before measurements and eliminated in the subsequent measurement. The concentration detection results indicate that when the background signal is eliminated, it can achieve a significant improvement in accuracy with a relative error of below 0.5% when the CO_2_ concentration is under 5000 ppm. The gas sensing method based on long-tune ln-WMS is valid for trace gas monitoring (weak absorbance) or in over-modulation situations.

## Figures and Tables

**Table 1:** Spectroscopic parameters of the selected transitions for CO_2_ and H_2_O near 2004 nm.

Gases	Wavelength (nm)	Line Strength (cm^−2^/atm)
**CO_2_ (Line A)**	2002.998	$3.07\times 10^{-2}$
**CO_2_ (Line B)**	2003.503	$3.21\times 10^{-2}$
**CO_2_ (Line C)**	2004.019	$3.28\times 10^{-2}$
**H_2_O (Line D)**	2002.829	$1.26\times 10^{-3}$

## Data Availability

The data that support the findings of this study are available from the corresponding author upon reasonable request.
